# One Health Genomic Surveillance at Human–Animal Interfaces in Rural Ghana Reveals Underreported Viruses of Zoonotic and Economic Concern

**DOI:** 10.3390/v18060644

**Published:** 2026-06-03

**Authors:** Julia E. Paoli, Nídia S. Trovão, Theophilus Odoom, Quaneeta Mohktar, Kwame Boamah Buabeng, Bright Adu, William Tasiame, Benita Anderson, Daniel Nana Yaw Tawiah-Yingar, Kuttichantran Subramaniam, Michael E. von Fricken, Gloria Ivy Mensah, Mario Mietzsch, Robert McKenna, Sherry Ama Mawuko Johnson, Carla N. Mavian

**Affiliations:** 1 Emerging Pathogens Institute, University of Florida, Gainesville, FL 32610, USA; 2 Department of Environmental and Global Health, University of Florida, Gainesville, FL 32610, USA; 3 Department of Pathology, Immunology and Laboratory Medicine, University of Florida, Gainesville, FL 32610, USA; 4 One Health Center of Excellence, College of Public Health and Health Professions, University of Florida, Gainesville, FL 32610, USA; 5 Department of Pathobiology, College of Veterinary Medicine, University of Illinois Urbana-Champaign, Urbana, IL 61802, USA; 6 Carl R. Woese Institute for Genomic Biology, University of Illinois Urbana-Champaign, Urbana, IL 61802, USA; 7 National Center for Supercomputing Applications, University of Illinois Urbana-Champaign, Urbana, IL 61802, USA; 8 Accra Veterinary Laboratory, Veterinary Services Directorate, Accra P.O. Box M161, Ghana; 9 School of Veterinary Medicine, College of Basic and Applied Sciences, University of Ghana, Legon, Accra P.O. Box LG139, Ghana; 10 Noguchi Memorial Institute for Medical Research, University of Ghana, Accra 00233, Ghana; 11 School of Veterinary Medicine, Kwame Nkrumah University of Science and Technology, Kumasi 00233, Ghana; 12 Veterinary Services Directorate, Ministry of Food and Agriculture, Accra P.O. Box M37, Ghana; 13 Department of Infectious Diseases & Immunology, College of Veterinary Medicine, University of Florida, Gainesville, FL 32610, USA; 14 Department of Biochemistry and Molecular Biology, College of Medicine, Center for Structural Biology, McKnight Brain Institute, University of Florida, Gainesville, FL 32610, USA; 15 Centre for Epidemic Response and Innovation, School for Data Science and Computational Thinking, Stellenbosch University, Stellenbosch 7600, South Africa

**Keywords:** One Health, metagenomic sequencing, Ghana, livestock, bats, phylogenetics, viral genomics

## Abstract

Under a One Health framework, viruses of veterinary and zoonotic importance pose significant threats to animal and human health, food security, and livelihoods, particularly in regions with intense human–animal interactions. In West Africa, despite recent advances in surveillance programs, important gaps remain in understanding viral diversity and cross-species transmission at wildlife–livestock interfaces. We conducted metagenomic surveillance to characterize viruses circulating across livestock, domestic animals, and wildlife in rural Ghana in 165 animals sampled across five regions. Viral RNA from serum and tissue samples was sequenced with the Illumina platform, and genomes were de novo assembled with MEGAHIT. Phylogenetic relationships were reconstructed using Bayesian approaches. We report the first genomic sequences of porcine parvovirus 3, canine parvovirus, rotavirus A genotype R16, and bovine hepacivirus subtype B from Ghana in over a decade. Phylogenetic analyses revealed intercontinental linkages between Africa and Europe for parvoviruses, persistence of hepacivirus lineages, and evidence of cross-species transmission for rotavirus. Notably, detection in apparently healthy animals highlights underrecognized circulation, gaps in vaccination effectiveness, trade-related biosecurity vulnerabilities, and the role of wildlife in viral maintenance and transmission. Our findings reveal dynamic viral diversity and connectivity across animal populations and ecological interfaces, emphasizing the fluid and interconnected nature of pathogen circulation within One Health systems. By integrating metagenomics and phylogenetics, this study provides a scalable framework for enhancing surveillance capacity, enabling the early detection of emerging threats and informing targeted strategies to mitigate zoonotic and economically important viral diseases in West Africa.

## 1. Introduction

Zoonotic viruses are a major global public health concern, with an estimated 75% of emerging infectious diseases originating from animal reservoirs and driven by anthropogenic, ecological, socioeconomic, and climatic factors [1]. A One Health approach integrating human, animal, and environmental surveillance is widely recognized as essential for the early detection and mitigation of zoonotic threats [2,3]. However, pathogen surveillance in livestock and wildlife remains underfunded relative to human health systems, and genomic surveillance capacity in animal populations is particularly limited in resource-constrained settings [2]. Strengthening animal-based surveillance is, therefore, critical for the early warning and prevention of emerging infectious diseases [2,4].

West Africa is a recognized hotspot for zoonotic disease emergence due to rapid population growth, increasing livestock densities, environmental change, and high levels of human–animal interaction [5,6,7,8]. Additional risk factors include bushmeat consumption, a culturally embedded practice in many rural communities [9]. Ghana has been actively implementing One Health strategies and is a member of the Global Health Security Agenda; however, recent Joint External Evaluations (2017 and 2025) have highlighted persistent gaps in zoonotic surveillance capacity and have recommended strengthened multisectoral systems [10,11]. Despite this, genomic data on viruses circulating in Ghanaian animal populations remain scarce across multiple host species.

Several viruses of veterinary importance also pose potential zoonotic and economic risks, including porcine parvovirus (PPV), canine parvovirus type 2 (CPV-2), bovine hepacivirus (BovHepV), and rotavirus A (RVA). PPV and CPV are small, single-stranded DNA viruses in the family *Parvoviridae*, with PPV1 being a major cause of reproductive failure in pigs and novel PPVs (PPV2–PPV8) increasingly reported worldwide, including in Africa [12,13,14]. CPV-2 is classified into antigenic variants 2a, 2b, and 2c, with CPV-2c associated with severe disease and vaccine breakthrough infections; however, genomic data from Ghana are lacking [15,16,17]. BovHepV, a hepatotropic positive-sense RNA virus in the family *Flaviviridae*, is genetically related to the hepatitis C virus (HCV) and has been reported in cattle globally, with evidence of infection in multiple animal species suggesting possible interspecies transmission [18,19,20]. Rotavirus A is a major cause of severe gastroenteritis in humans and animals, with a segmented genome that facilitates reassortment [21,22,23]. RVA has been detected in bats, including partial genomes previously reported from the straw-colored fruit bat (*Eidolon helvum*) in Ghana [24].

Here, we applied a One Health metagenomic approach to investigate viral diversity in wildlife (bats), livestock (cattle and pigs), and companion animals (dogs) in Ghana. We report the first genomic detection of multiple viral pathogens circulating at the human–animal interface in the country, addressing critical surveillance gaps and providing baseline genomic data to support early warning systems for emerging zoonotic threats.

## 2. Materials and Methods

### 2.1. Study Area and Sample Collection

**Figure 1:**
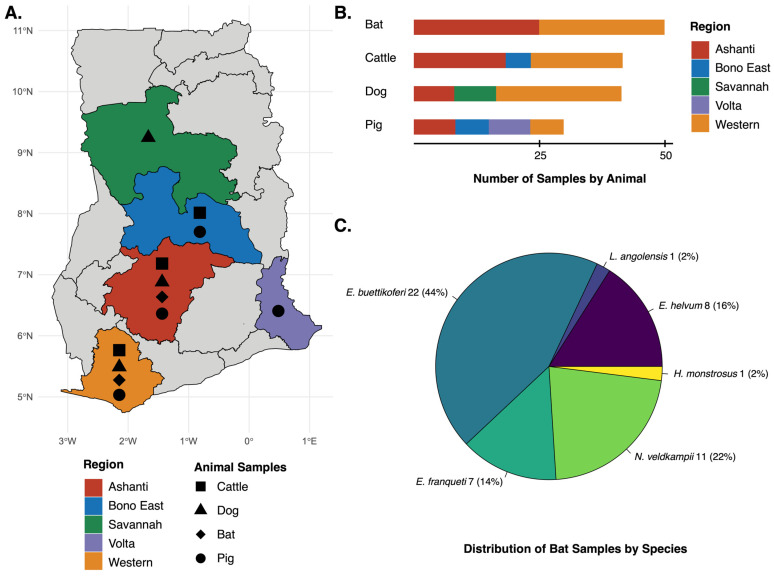
Geographic distribution of sampling locations and animal samples collected across Ghana: (**A**) Map of Ghana showing regions where sampling occurred. The Ashanti, Bono East, Savannah, Volta, and Western Regions are highlighted. Symbols within each region indicate the animal type sampled. (**B**) Number of total samples collected by animal type (n = 166). Colors indicate region of sampling. (**C**) Distribution of bat species sampled, showing species composition, sample number, and percentage of total bat samples collected (n = 50).

Wildlife (bats) were sampled in 2022 while livestock (cattle and pigs) and companion animals (dogs) were sampled in 2023 across communities in five of Ghana’s 16 administrative regions: Ashanti, Bono East, Savannah, Volta, and Western (Figure 1, Table 1). Bat sampling sites were selected based on the incidence of Marburg virus disease in humans in 2022 [25] and intensive mining and farming activities occurring in the area [8]. Bats were captured using mist nets and harp traps, and captured bats were temporarily placed in cotton bags before transferring to mesh holding cages for morning processing following the standard operating procedure of the Institutional Animal Care and Use Committee [26]. Bats were identified in the field by trained personnel using morphological keys [27,28,29]. Sex was determined by external genital morphology, and age class (juvenile, sexually immature adult, or adult) was assigned based on morphological traits (Table S1). Reproductive status and clinical signs were also recorded. Bats were euthanized via cervical dislocation [30]. Spleen, liver, kidneys, and intestines were aseptically sampled from each bat, placed into separate labeled cryotubes, and transported in liquid nitrogen to the Accra Veterinary Laboratory.

Sampling sites for livestock and dogs were selected based on animal density and associated risk factors, including hunting practices and extensive farming systems [8]. Serum samples from cattle, pigs, and dogs were collected and processed as previously described [8]. Briefly, blood (5 mL) was collected from livestock (jugular vein) and dogs (cephalic vein) into serum separator tubes and transported in liquid nitrogen to the Accra Veterinary Laboratory. Samples were centrifuged (2500 rpm, 3 min), and sera were aliquoted and stored at −20 °C until testing. Age, sex, weight, reproductive status, and clinical signs were also recorded for these animals (Table S1).

Sampling was conducted during both dry and rainy seasons, which differ in timing between northern and southern Ghana [8,31]. Northern Ghana experiences a rainy season from March to September and a dry season from November to June, whereas southern Ghana has two rainy seasons (April–June and September–November) separated by a dry season (December–March).

All personnel were trained and wore appropriate PPE during sample collection. All animal sampling was approved by the University of Ghana Animal Care and Use Committee (UG-IACUC 032/22-23), and the study was conducted in accordance with the relevant guidelines and regulations. All dissections were performed by board-certified.

### 2.2. Viral Extraction and cDNA Synthesis

For the cattle, pig, and dog serum samples, viral RNAs were extracted from 800 µL of serum using the Quick-RNA Viral Kit (Zymo Research, Irvine, CA, USA), according to the manufacturer’s protocol for serum samples. The bat spleen, liver, kidney, and intestine samples were pooled and then homogenized using an electric homogenizer; afterwards, RNA was extracted using the Quick-RNA Viral Kit (Zymo Research, Irvine, CA, USA), according to the manufacturer’s protocol for tissue samples. In addition to isolating viral RNA, this Zymo kit co-purifies select DNA viruses, including parvoviruses. After extraction, cattle, pig, and dog samples were grouped by species, collection site, and date for library preparation, with an average of four samples per group (n = 1–5) (Table S1). Equal volumes of extracted nucleic acid from each sample were combined to create a final volume of 8 µL per pool. The bat specimens were sequenced individually and not pooled.

To prepare the complementary DNA (cDNA), RNA was processed according to the previously described RAPIDprep assay for the following steps: genomic DNA removal, ribosomal RNA depletion (rRNA), double-stranded cDNA synthesis, and cDNA purification [32,33]. To reduce reagent consumption, two modifications were made to the original protocol as previously described, which yielded comparable results to the standard assay [34,35]. For the rRNA removal step, the QIAseq FastSelect (Qiagen, Hilden, Germany) dilution mixture was prepared at a 1:1:8 ratio by combining 1 µL of 5S/16S/23S rRNA removal reagent, 1 µL of HMR (human/mouse/rat) depletion reagent, and 8 µL of nuclease-free water. The Sequenase enzyme dilution used for second-strand synthesis was prepared to provide 1 unit per reaction, corresponding to 0.08 µL of Sequenase Version 2.0 DNA Polymerase (Thermo Fisher Scientific, Waltham, MA, USA) mixed with 0.92 µL of nuclease-free water.

### 2.3. Illumina Library Prep and Sequencing

Illumina sequencing libraries were prepared using the NEBNext Ultra II FS DNA Library Prep Kit (New England Biolabs, Ipswich, MA, USA) and NEBNext Multiplex Oligos for Illumina Dual Index Primers Set 1 (New England Biolabs, Ipswich, MA, USA) following the protocol for inputs ≤ 100 ng. In place of AMPure XP Beads (Beckman Coulter, Brea, CA, USA), Omega Bio-tek Mag-Bind Total Pure NGS beads (Omega Bio-tek, Norcross, Georgia, USA) were used for all cleanup steps. Library concentrations were measured on a Qubit 4 Fluorometer (Thermo Fisher Scientific, Waltham, MA, USA) using the Qubit dsDNA HS Assay Kit (Thermo Fisher Scientific, Waltham, MA, USA). Library quality and fragment size were assessed with the Agilent 4150 TapeStation system (Agilent Technologies, Santa Clara, CA, USA). We conducted two rounds of sequencing: the first round consisted of the cattle and dog samples pooled together, while the second round comprised the bat and pig samples pooled together. For each round of sequencing, individually barcoded libraries were pooled in equimolar concentrations to construct the final library. Sequencing was conducted on the NextSeq 1000 platform using the P2 Reagents (100 cycles) v3 cartridge at a 2 × 50 bp read length (Illumina, San Diego, CA, USA).

### 2.4. NGS Data Analysis

FASTQ files were trimmed with Trimmomatic v0.39 [36] to remove adapters and low-quality bases. De novo assembly was performed with MEGAHIT v1.1.4 [37], and resulting contigs were compared to the NCBI BLAST nt/nr databases and the InterPro Database to investigate taxonomic identity [38,39]. Sequencing depth and genome coverage were calculated using Bowtie2 v2.5.4 [40] and Samtools v1.20 [41] by aligning reads to de novo-generated genomes (Table 2). Sequencing reads were deposited in the NCBI Sequence Read Archive (SRA) under Project ID: PRJNA1455158. Genome sequences were deposited to GenBank with the following accessions: PPV3 (PX310535.1); CPV-2 (PZ284984.1 and PZ284985.1); BovHepV (PZ284983.1); and RVA segment 1 (PZ284986.1).

### 2.5. Dataset Generation and Recombination Analysis

For each virus of interest, a background dataset was constructed by retrieving publicly available complete genome sequences and associated metadata from the NCBI Virus database [42]. A list of GenBank accession numbers and metadata of all reference sequences included is listed in Supplementary Table S2, and the search terms for each are given in Supplementary Table S3. For all viruses of interest, multiple sequence alignments of the study genomes with comparative reference sequences were generated using MAFFT v7.520 [43], and we trimmed the 5′ and 3′ ends. Alignments were then manually refined in AliView v1.28 [44]. We removed redundant sequences, defined as those sharing identical collection date, country of origin, and nucleotide sequence, using a custom python script (https://github.com/jlcherry/CAMPI; accessed on 11 August 2025).

Recombination screening was carried out using RDP5 under default parameters for linear genomes [45]. Recombination events were considered supported if detected by at least six out of the following seven methods with *p*-values < 0.05: RDP, GENECONV, Chimaera, MaxChi, BootScan, SiScan, and 3Seq. Identified recombinant regions were excluded to generate recombination-free alignments.

### 2.6. Pairwise Genetic Distance Analysis

Pairwise genetic distances were calculated from nucleotide sequence alignments using Molecular Evolutionary Genetics Analysis version 11 (MEGA11) [46] and are presented in Supplementary Table S4. Both the number of nucleotide differences and *p*-distances were estimated under the following conditions: gamma-distributed rates with a shape parameter of 4.0; 100 bootstrap replicates for variance assessment; treatment of gaps as complete deletions; and inclusion of all codon positions (1st, 2nd, 3rd) along with non-coding regions.

### 2.7. Bayesian Phylodynamic Analysis

For each virus of interest, the temporal signal within the dataset was evaluated using TempEST v1.5.3 [47] on maximum-likelihood phylogenies reconstructed in IQ-TREE v2.2.7 [48], with the best-fit nucleotide substitution model selected according to the Bayesian Information Criterion (BIC) and branch support assessed via 2000 ultrafast bootstrap replicates. Bayesian phylogenetic analysis was performed using the Bayesian Evolutionary Analysis Sampling Trees (BEAST) v10.5.0 [49]. Evolutionary parameters were estimated with the general time reversible (GTR) model of nucleotide substitution, empirical base frequencies, and gamma-distributed site heterogeneity. The relaxed molecular clock model [50] was used in combination with either the Hamiltonian Monte Carlo SkyGrid [51] population prior or a constant population prior, depending on the data. Default settings were applied for all remaining priors and operators. MCMC chains were run for at least 500 million generations, with sub-sampling every 50,000 iterations across three independent replicates. Convergence and adequate sampling were assessed using Tracer v1.7.2 [52], ensuring all parameters had effective sample sizes (ESSs) greater than 200 after discarding at least the initial 10% of generations as burn-in. A maximum clade credibility (MCC) tree was constructed in TreeAnnotator v1.10.5, summarizing the posterior distribution of trees with median node heights and applying a 10% burn-in. The resulting MCC tree was visualized in R Studio using the ggtree v3.2.1 package [53]. The evolutionary rate for each virus, estimated from the slope of the best-fit regression line in TempEst, is provided in Supplementary Table S5.

### 2.8. Capsid Model Generation and Visualization

AlphaFold3 was utilized to obtain models of the viral protein 1 unique region (VP1u) of PPV3 and CPV-2, the envelope proteins E1 and E2 of BovHepV, and the RNA-dependent RNA polymerase (RdRp) of RVA [54]. For the generation of icosahedral capsids, https://foldavirus.org/ (Accessed on 24 April 2026) was used. The models were visualized and colored using ChimeraX [55]. Amino acid sequence differences were identified with Clustal W.

### 2.9. Map Construction

GPS locations of the animal samples were recorded at collection. Administrative boundary shapefiles were obtained from the Global Administrative Areas (GADM) dataset and accessed on 9 March 2026, via the geodata R package [56]. These data are freely available for academic and non-commercial use. The map was visualized in R using the packages ggplot2 [57], terra [58], readr [59], dplyr [60], tidyr [61], and sf [62].

## 3. Results

### 3.1. Animal Collection

During the study period, a total of 166 animals from five regions were sampled, comprising cattle (n = 43; 26%), pigs (n = 31; 19%), dogs (n = 42; 25%), and bats (n = 50; 30%) (Figure 1, Table 1). The bat samples comprised six species, with the following sample sizes: *Eidolon helvum* (n = 8; 16%), *Epomops buettikoferi* (n = 22; 44%), *Epomops franqueti* (n = 7; 14%), *Hypsignathus monstrosus* (n = 1; 2%), *Lissonycteris angolensis* (n = 1; 2%), and *Nanonycteris veldkampii* (n = 11; 22%) (Table 1, Figure 1). Most animals were drawn from the Western Region (n = 76; 46%), followed by Ashanti (n = 61; 37%), Bono East (n = 11; 7%), Savannah (n = 9; 5%), and Volta (n = 9; 5%) (Table 1, Figure 1). For the cattle, pigs, and dogs, overall, the animals consisted of adults (n = 72; 62%), young adults (n = 16; 14%), young animals (n = 28; 24%), and females (n = 72; 62%) (Table S1). The bats consisted of adults (n = 36; 72%), subadults (n = 13; 26%), juveniles (n = 1; 2%), and females (n = 28; 56%) (Table S1).

### 3.2. First Genomic Detection of Porcine Parvovirus 3 (PPV3) and Canine Parvovirus 2 (CPV-2) in Ghana

We identified a full-genome sequence of PPV3 from a male, two-year-old Large White cross pig sampled in the Volta Region on 18 July 2023 (ID: P32) (Table S1). The pig came from an intensive farming system flock comprising 150 pigs in total. The owner of the farm also kept sheep and poultry as livestock, in addition to farm dogs and cats. De novo assembly produced a single viral contig of 5208 bp (contig name k141_88414; GenBank ID PX310535.1), which shared 99% nucleotide identity with a PPV3 sequence from a domestic pig sampled in China in 2018 (MZ577031.1) according to BLAST analysis (Table 2). Sequencing yielded high coverage of the PPV3 genome, with an average read depth of 1352X across the full genome (Table 2). We found no evidence of recombination in PX310535.1. Pairwise genetic distance analysis revealed the PPV3 genome to be highly conserved across the complete coding region (Table S4). The Ghanaian virus was closely related to sequences sampled from wild boar in Romania in 2010 (*p*-distance = 0.004; SE = 0.001) and in Italy in 2016 (*p*-distance = 0.003; SE = 0.001).

**Figure 2:**
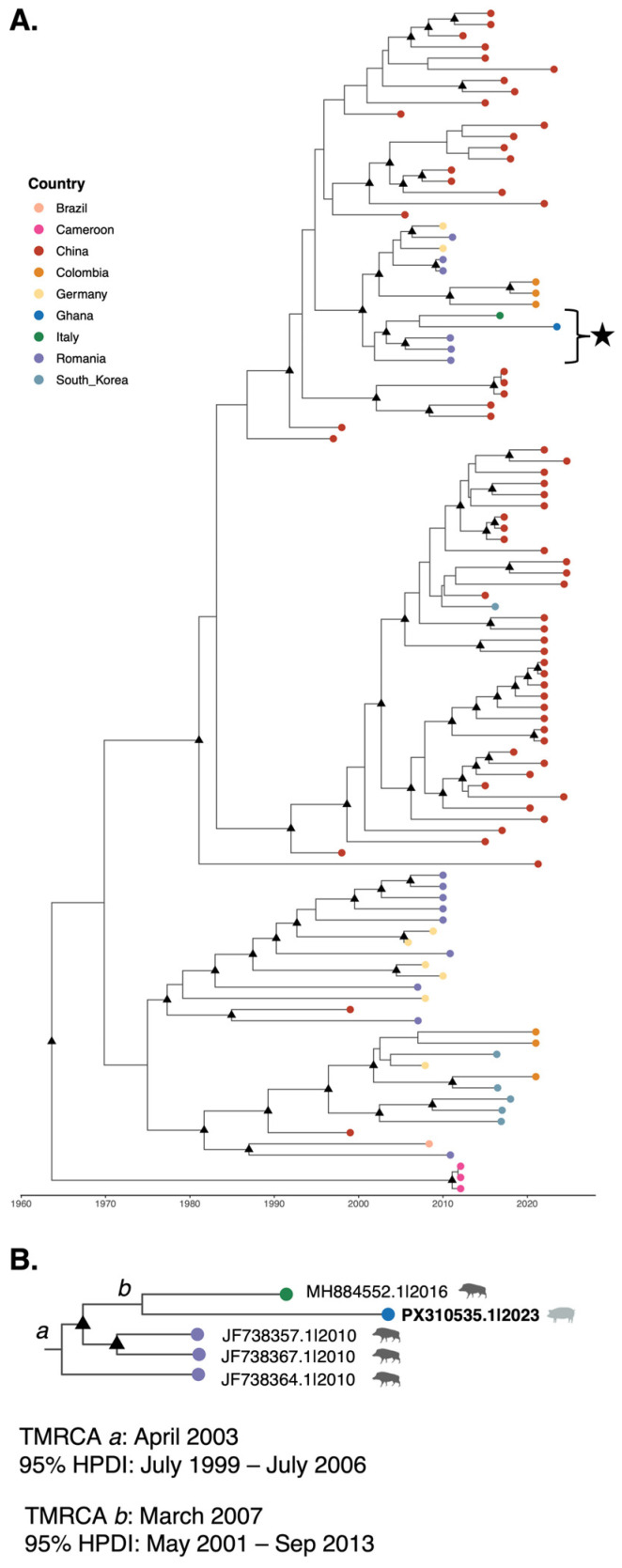
Time-scaled maximum clade credibility (MCC) tree estimated from complete coding region of PPV3 sequences: (**A**) MCC tree inferred using the uncorrelated relaxed clock and Hamiltonian Monte Carlo SkyGrid implemented in BEAST X v10.5.0. Tips are colored according to country of origin. Black triangles at internal nodes indicate branches supported by posterior probability > 0.9. The subclade of interest containing the sequence from Ghana generated in this study, PX310535.1, is highlighted with a black star. (**B**) Zoomed view of the highlighted subclade shown in panel A. For selected nodes, *a* and *b*, the time to most recent common ancestor (TMRCA) and 95% highest posterior density intervals (HPDIs) are given. Node *a* represents the most recent common ancestor of PX310535.1 and wild boar sequences of Romanian and Italian origin. Node *b* represents the common ancestor of PX310535.1 and MH884552.1 from a wild boar sampled in Italy. Viruses are labeled by GenBank ID and date of collection; host is indicated by silhouette of wild boar in dark gray or domestic pig in light gray.

Bayesian phylodynamic reconstruction of global PPV3 sequences estimated the origin of emergence of PPV3 to be around August 1963 (95% highest posterior density interval [HPDI]: March 1943–March 1981) (Figure 2). Our study sequence, PX310535.1, clustered within a monophyletic and well-supported clade (posterior probability > 0.99) comprising sequences collected from domestic pigs in Colombia, Germany, and Romania, as well as wild boars from Romania and Italy (Figure 2). Within this clade, there were two sister subclades: subclade 1 comprised sequences from both Europe and South America, while subclade 2 consisted of sequences from Europe and our study sequence from Ghana. PX310535.1 clustered in a lineage comprising sequences from wild boar sampled in Romania in 2010 (JF738364.1, JF738367.1, and JF738357.1) and in Italy in 2016 (MH884552.1), with an estimated time to the most recent common ancestor (TMRCA) in April 2003 (95% HPDI: July 1999–July 2006) (Figure 2). Within this lineage, our study sequence formed a subclade with MH884552.1, although moderately well supported (posterior probability = 0.87), with an estimated TMRCA in March 2007 (95% HPDI: May 2001–September 2013) (Figure 2). This topology was consistent with the maximum-likelihood phylogeny (Figure S1). The clustering of PPV3 sequences from domestic pigs with sequences from wild boars may indicate transmission pathways between wild and domesticated animals, including between wild pigs in Europe and domesticated pigs in Ghana. The long branch length of PX310535.1 could indicate unsampled diversity among Ghanaian pigs. Notably, the Ghana sequence clustered separately from the only other available complete coding region genomes from the African continent, which were sampled in Cameroon from domestic pigs in 2012 (KF225550.1, KF225549.1, and KF225548.1) and formed a distinct lineage (Figure 2).

The capsid gene of the Ghanaian PPV3 genome potentially expresses two overlapping viral proteins (VPs). This is comparable to other viruses of the *Parvovirinae* and genus *Tetraparvovirus* [63]. VP1 is composed of 925 amino acids (aas) and contains the conserved phospholipase A2 (PLA_2_) domain plus a potential additional domain in its N-terminal VP1 unique (VP1u) region based on AlphaFold3 predictions (Figure S2). Similar domains in VP1u have been observed for other parvoviruses, such as Parvovirus B19, and act as receptor-binding domains [64]. VP2 contains 555 aas and possesses a short glycine-rich motif at its N-terminus as previously described for other parvoviruses [63] (Figure S2). Near the C-terminus, a basic patch is located, which in AlphaFold models is predicted to be located at the interior side of the capsid near the 2-fold symmetry axis and could potentially act as a DNA binding site. This predicted capsid structure shows the conserved features observed in other parvovirus capsids, such as channels at the 5-fold symmetry axis and three protrusions surrounding the 3-fold axis (Figure S2). Similar to clustering patterns observed for the complete coding region, phylogenetic analysis clusters the Ghanaian PPV3 VP1 sequence in a clade with wild boar samples sampled in Italy and Romania (Figure S2). Comparisons of the amino acid sequence show only one aa difference to the Italian isolate and five to eight aa differences to the Romanian viruses (Figure S2).

A globally distributed virus, PPV3, has been detected in domestic and wild pigs, with previous reports of PPV3 in Africa described among domestic pigs in the Democratic Republic of the Congo [65], South Africa [66], Nigeria [67], and Cameroon [68]. Among domestic pigs, reported PPV3 prevalence differs by geographic region, detection method, and age, with a wide range of estimates in the literature (3.1–52%) [12,69,70]. In our study, PPV3 was detected in the serum of one adult male pig with no reported clinical symptoms. The low detection rate among Ghanaian pigs compared to other studies in African pig populations may be a reflection of testing apparently healthy animals, whereas some other studies with higher prevalence rates may have tested sick or deceased pigs or obtained higher detection rates from tissue samples [65,68,71,72,73]. The lack of previous PPV3 reports in Ghana suggests cryptic transmission among pigs. The clustering of PX310535.1 with European wild boar-derived sequences, along with an inferred divergence dating back to approximately 2007, suggests that this lineage may have been introduced into Ghana well before its detection. The extended branch length of PX310535.1 is consistent with a period of unobserved evolution, potentially reflecting gaps in surveillance.

We identified one complete genome (contig name k59_937; GenBank ID PZ284984.1; 5034 bp) and one near-complete genome (contig name k59_26528; GenBank ID PZ284985.1; 4953 bp; 98%) of CPV-2 from companion dogs with no clinical symptoms sampled in the Western and Savannah Regions, respectively (Table 2). These sequences were derived from two separate pools (Western = 3D; Savannah = 1D), each comprising four dog samples. None of the sampled dogs exhibited clinical symptoms; all were local breeds; and each pool included both female and male dogs (Table S1). Both genomes were sequenced at a high depth of coverage, 607X for PZ284985.1 and 302X for PZ284984.1, and showed high nucleotide identity to previously reported CPV-2c strains from Asia (Table 2). No evidence of recombination was detected in either sequence. Pairwise distance analysis demonstrated that the two study sequences were identical across the coding region, indicating a high degree of conservation of CPV-2 circulating across administrative regions in Ghana (Table S4). Both sequences showed the greatest similarity to CPV-2c strains sampled in China between 2020 and 2021, sharing 99% nucleotide identity across the coding region (mean *p*-distance = 0.002; mean SE = 0.001) (Table S4).

**Figure 3:**
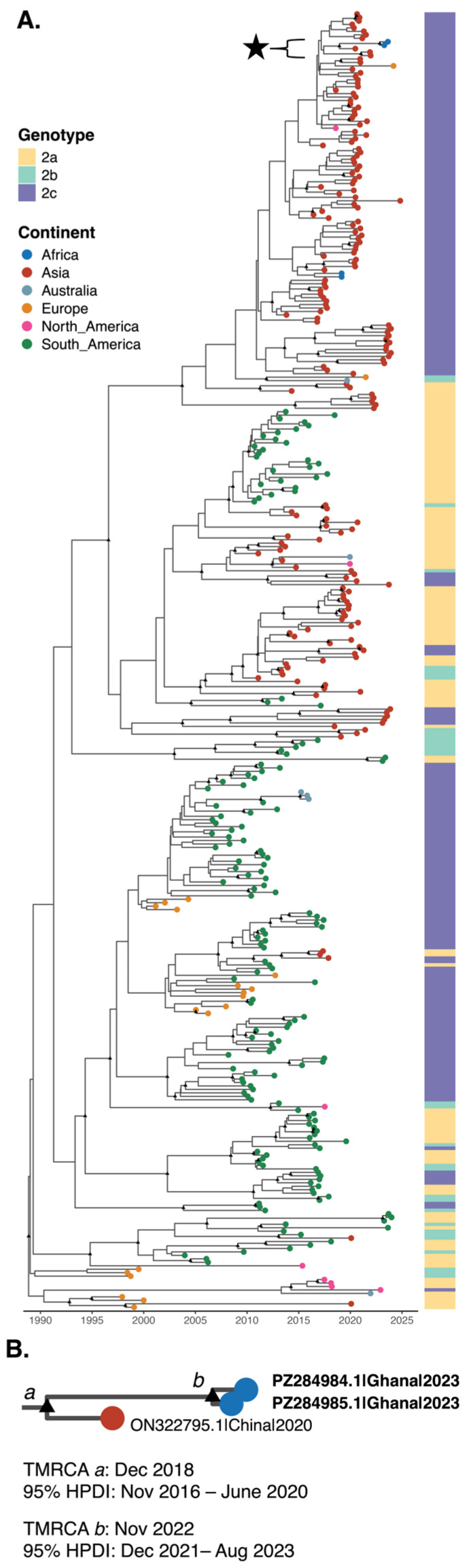
Time-scaled maximum clade credibility (MCC) tree estimated from CPV-2 complete coding region sequences: (**A**) MCC tree inferred using the uncorrelated relaxed clock and Hamiltonian Monte Carlo SkyGrid implemented in BEAST X. Tips are colored according to continent of origin. The heat map is colored according to genotype. Black triangles at internal nodes indicate branches supported by posterior probability > 0.9. The subclade containing the sequences from Ghana generated in this study, PZ284984.1 and PZ284985.1, is highlighted with a black star. (**B**) Zoomed view of the study sequences subclade shown in panel A. For selected nodes, *a* and *b*, the time to most recent common ancestor (TMRCA) and 95% highest posterior density intervals (HPDIs) are given. Node *a* represents the most recent common ancestor of our study sequences and ON322795.1. Node *b* represents the TMRCA of study sequences. Viruses are labeled by GenBank ID, country of origin, and date of collection.

Bayesian phylogenetic reconstruction confirmed both study sequences clustering within the CPV-2c clade (Figure 3), specifically within an international subclade containing strains sampled from Spain, Gabon, China, and Canada. Notably, the Ghanaian sequences, PZ284985.1 and PZ284984.1, were positioned on relatively long branches compared to other members of this subclade, suggesting the presence of unsampled viral diversity circulating within Ghana. Within this group, the study sequences clustered with strong support (posterior probability = 0.99) alongside a sequence from a dog collected in China in 2020 (ON322795.1), with an estimated TMRCA of December 2018 (95% HPDI: November 2016–June 2020). The TMRCA for the two Ghanaian sequences was estimated to be November 2022 (95% HPDI: December 2021–August 2023). The maximum-likelihood phylogeny showed consistent placement of both Ghanaian sequences within the CPV-2c clade (Figure S3).

On the protein level, the Ghanaian CPV-2c strain is identical or has two aa differences in the NS protein compared to the Chinese 2a and the Hungarian 2b strains, respectively. For the capsid proteins, the differences are slightly higher, with three or seven aa differences in VP1, resulting in a 99.6% and 99.0% sequence identity (Figure S4). All differences in VP2 are situated at the capsid surface and cluster around the 3-fold protrusion. None of the CPV-2c VP2 residue changes observed in the study sequences affect the previously described binding site for the transferrin receptor [74]. However, the Q370R change in the CPV-2b and -2c strains was described to enhance pathogenicity [75]. Position 426 differs in the three strains and was suggested to play a role in the antigenic drift of CPV, potentially leading to reduced detection of host antibodies [76].

### 3.3. First Detection of Bovine Hepacivirus (BovHepV) Subtype B in Ghanaian Cattle Since 2011

We detected a complete BovHepV genome (contig name k59_164; GenBank ID PZ284983.1; 8802 bp; 99%) in a pooled sample from four cattle collected in the Ashanti Region in March 2023 (Table 2). All cattle included in the pool (Pool ID: 6C) were adult females with no reported clinical signs (Animal IDs: KMC16, KMC27, KMC42, and FMC47) (Table S1). Based on the BLAST analysis, the study sequence shared the highest nucleotide identity (91%) with the reference BovHepV strain NC_026797.1, which was previously identified in cattle in Ghana in 2011 (Table 2). Sequencing of the study genome yielded a high average coverage depth of 120X. No evidence of recombination was detected in the study sequence. Pairwise genetic distance analysis indicated that PZ284983.1 exhibited the greatest identity to subtype B sequences, all of which were collected in Ghana in 2011 (mean *p*-distance = 0.094; SE = 0.004), compared to other subtypes, including subtype C sequences from Ghana (2011) and Uganda (2018) (mean *p*-distance = 0.170; SE = 0.005) (Table S4) [77,78,79].

**Figure 4:**
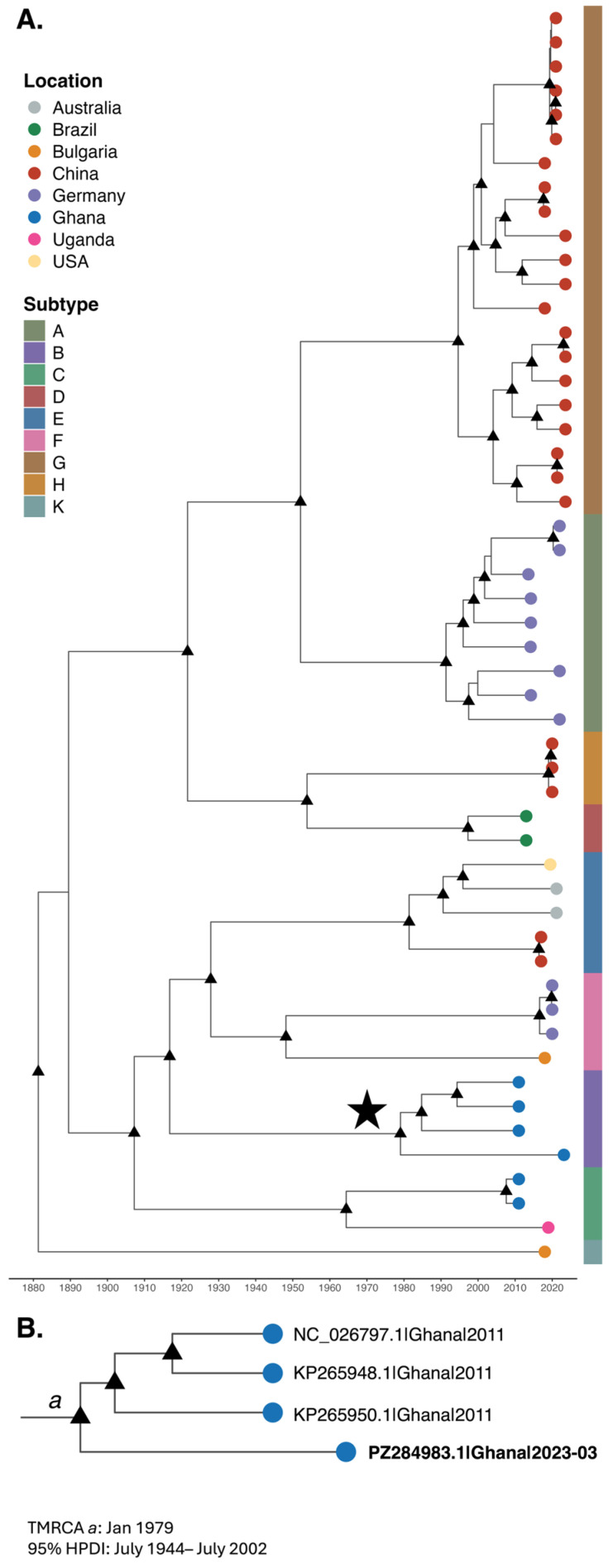
Time-scaled maximum clade credibility (MCC) tree estimated from complete coding region of BovHepV genotype 1 sequences: (**A**) MCC tree inferred using the uncorrelated relaxed clock and constant coalescent implemented in BEAST X. Tips are colored according to country of origin. The heat map is colored according to subtype. Black triangles at internal nodes indicate branches supported by posterior probability > 0.9. The subtype B subclade containing the sequence from Ghana generated in this study, PZ284983.1, is highlighted with a black star. (**B**) Zoomed view of the subtype B subclade shown in panel A. For node *a*, the time to most recent common ancestor (TMRCA) and 95% highest posterior density interval (HPDI) are given. Node *a* represents the most recent common ancestor of PZ284983.1 and previously published subtype B sequences from Ghanaian cattle collected in 2011. Viruses are labeled by GenBank ID and date of collection.

Two subtypes of BovHepV genotype 1, subtypes B and C, have been reported to circulate in Ghana [77]. Bayesian phylogenetic reconstruction placed the study sequence, PZ284983.1, within a well-supported (posterior probability = 0.99) monophyletic subclade comprising BovHepV subtype B sequences collected from cattle in Ghana (Figure 4). All currently available subtype B sequences originate from Ghanaian cattle, indicating a geographically restricted lineage. The estimated TMRCA of the subtype B subclade of January 1979 (95% HPDI: July 1944–July 2002) supports sustained circulation of this subtype within the region over an extended period. Although additional subtype B sequences have been reported from cattle in Italy in 2014, these are partial genomes and were therefore not included in our analysis [80]. Nonetheless, the Italian subtype B NS3 and 5′ UTR sequences are closely related to the Ghanaian subtype B sequences identified in 2011 [80]. The maximum-likelihood phylogeny showed consistent clustering of the study sequence within the subtype B clade (Figure S5).

Phylogenetic analysis of the E1 and E2 sequences supports placement of PZ284983.1 within subtype B (Figure S6). Compared to other Ghanaian subtype B sequences (NC_026797.1, KP265928.1, and KP265950.1), the study sequence differed in the E1 domain by three to five amino acids, and in the E2 domain by two to four amino acids (Figure S6). A limited amount of literature is available on the BoVHepV envelope proteins; however, compared to the closely related HCV, the E1 protein is of equivalent length while the E2 protein is 96 aas shorter [81]. Neutralizing antibody sensitivity to HCV is governed by molecular and structural features of E1/E2, and thus, the heterodimer may be a good vaccine design target [82]. Further studies of BovHepV E1/E2 could be beneficial for vaccine development.

### 3.4. First Detection of Rotavirus A (RVA) Segment 1 Genotype R16 in Ghana

We obtained a near-complete RVA segment 1 genome (contig name k59_84; GenBank ID PZ284986.1; 3143 bp; 95%) out of a total of 11 RVA segments. PZ284986.1 was sequenced with an average depth of 30X from an *E. helvum* subadult male (Animal ID: FK39) sampled in the Ashanti Region on 22 October 2022, which was the only one of six *E. helvum* specimens to test positive for RVA (Table S1). This represents a previously undocumented host–location association. No viral genomes were detected from the 19 other bats sampled in Ashanti (species: *E. buettikoferi*, *E. franqueti*, *L. angolensis*, and *N. veldkampii*), nor from the 25 bats sampled in the Western Region (species: *E. buettikoferi*, *E. franqueti*, *E. helvum*, *H. monstrosus*, and *N. veldkampii*). The Ghanian RVA genome, PZ284986.1, shared 98% nucleotide identity with an RVA segment 1 genome of genotype R16 (KX268776.1) sampled from *E. helvum* in Cameroon in December 2013 (Table 2). No evidence of recombination was detected within the Ghanaian RVA genome. Pairwise distance analysis of PZ284986.1 with reference RVA sequences revealed our study sequence shared the closest nucleotide identity with R16 genotype sequences sampled from *E. helvum* in Cameroon in 2013 (KX268776.1; *p*-distance = 0.018; SE = 0.004) [83] and in Saudi Arabia in 2012 (KX420939.1; *p*-distance = 0.027; SE = 0.005) (Table S4) [84]. PZ284986.1 was more distantly related to the only other available RVA segment 1 sequence from Ghana, belonging to genotype R15, sampled from *E. helvum* in 2009 (MN567261.1; *p*-distance = 0.060; SE = 0.007) (Table S4) [24]. According to the ICTV criteria, segment 1 sequences with ≥83% nucleotide identity are assigned to the same genotype [85]. These results, together with phylogenetic reconstruction, strongly support the classification of PZ284986.1 as an R16 genotype.

**Figure 5:**
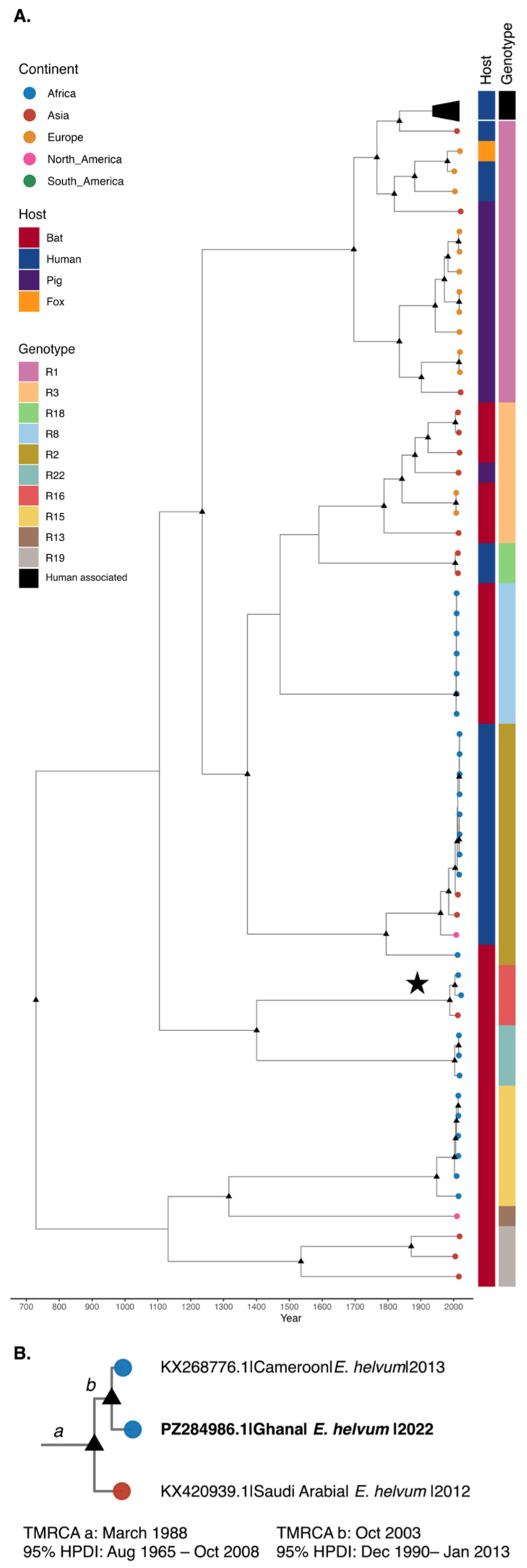
Time-scaled maximum clade credibility (MCC) tree estimated from RVA segment 1 sequences: (**A**) MCC tree inferred using the uncorrelated relaxed clock and Hamiltonian Monte Carlo SkyGrid implemented in BEAST X. Tips are colored according to continent of origin. The heat maps are colored according to host and genotype. Black triangles at internal nodes indicate branches supported by posterior probability > 0.9. The genotype R16 subclade containing the sequence from Ghana generated in this study, PZ284986.1, is highlighted with a black star. A clade containing human sequences of human-associated genotypes is collapsed, as indicated by trapezoid symbol. (**B**) Zoomed view of the genotype R16 subclade shown in panel A. For selected nodes, *a* and *b*, the time to most recent common ancestor (TMRCA) and 95% highest posterior density intervals (HPDIs) are given. Node *a* represents the most recent common ancestor of our study sequences and *E. helvum* R16 sequences. Node *b* represents the common ancestor of study sequence PZ284986.1 and KX268776.1 sampled in Cameroon. Viruses are labeled by GenBank ID, host, and date of collection.

The study sequence, PZ284986.1, clustered in a monophyletic, well-supported (posterior probability = 0.99) subclade comprising *E. helvum* sequences of segment 1 genotype R16 sampled from Cameroon in 2013 (KX268776.1) and Saudi Arabia in 2012 (KX420939.1) (Figure 5) [24,83]. The estimated TMRCA of the R16 lineage was March 1988 (95% HPDI: August 1965–October 2008) (Figure 5). Within this lineage, the Ghanaian sequence clustered more closely with the sequence from Cameroon, with an estimated TMRCA of October 2003 (95% HPDI: December 1990–January 2013) (Figure 5). The only other publicly available RVA segment 1 sequence from Ghanaian bats was collected from *E. helvum* in 2009 in Kumasi, Ashanti Region, and belongs to the R15 genotype (Figure 5) [24]. Additionally, an included human sequence collected from Ghana in 2015 (ON737599.1) clustered separately from both bat-borne Ghanaian sequences in the human-associated R1 genotype clade (Figure S7). Consistent with prior studies of RVA in bats [83], the infected individual in our study did not exhibit clinical signs of disease, although the recovery of segment 1—which encodes the viral polymerase gene—is indicative of viral replication and active infection. To our knowledge, this represents the first report of the R16 genotype in Ghana. The maximum-likelihood phylogeny supported the placement of PZ284986.1 within the R16 genotype (Figure S8).

The RdRp structure is highly conserved among R16 genotype sequences (Figure S9). Most aa differences between the study sequence and the isolates from Cameroon (KX268776.1) and Saudi Arabia (KX420939.1) were identified in the N-terminal domain, with six aa differences relative to KX268776.1 and nine aa differences relative to KX420939.1 (Figure S9). In the bracelet domain, the study sequence had a single aa difference relative to KX420939.1 (Figure S9). The N-terminal domain regulates the genome and may recruit transcription factors, thus any aa changes may alter viral replication [86].

## 4. Discussion

Ghana has experienced multiple infectious disease outbreaks of zoonotic origin, including Marburg virus disease [87], Mpox [88], and Lassa fever [89], highlighting the increasing importance of integrated surveillance at the human–animal–environment interface. Livestock and companion animals can act as bridging hosts between wildlife and humans, particularly in settings with frequent cross-species interactions [90], including where cultural and farming practices, hunting, and bushmeat consumption increase opportunities for cross-species transmission [8]. In this context, our study demonstrates how genomic surveillance across multiple host species can enhance the detection of circulating viruses and reveal diversity not captured by routine diagnostics [91].

The observed genetic links between viruses circulating in Ghana and those from Europe, the Middle East, and other parts of Africa highlight the role of transboundary processes—including livestock trade, animal movement, and ecological connectivity—in shaping viral circulation. These findings support the need for strengthened monitoring of animal movement and imported breeding materials, alongside improved biosecurity. For example, our findings support the need for targeted screening of imported semen and livestock, as well as monitoring of artificial insemination practices. Although PPV3 has not been documented in semen, other parvoviruses (e.g., PPV4 and PPV7) have been detected in this medium, suggesting a plausible route of introduction [92]. Unregulated movement of genetic material represents a plausible pathway for viral introduction. Anecdotal reports indicate that pig semen may be informally sourced from Europe and South Africa for breed improvement on private farms. Although such practices are difficult to verify, our findings may be consistent with this possibility, particularly in cases where investigations of reproductive failure have ruled out more common pathogens, such as *Brucella* spp. and *Leptospira* spp.

Detection of BovHepV is notable, as emerging evidence indicates it can infect species beyond cattle, including deer, sheep, and wild boar [18,20,93]. Its identification in blood-feeding ticks further suggests a possible role for arthropod-mediated transmission [94]. In mixed-species farming systems, particularly those involving cattle and swine, monitoring for signs of liver disease and limiting interspecies contact may help mitigate spread. Together, these observations highlight the expanding host range and transmission complexity of BovHepV in multi-host environments.

The identification of circulating variants in companion animals, such as CPV-2c, raises important considerations for vaccine effectiveness and coverage in local contexts. First reported in Ghana in 2017, CPV-2 continues to be detected in clinically affected dogs through 2023–2024, indicating persistent endemic circulation despite vaccination efforts [15,16]. This likely reflects incomplete vaccination, improper administration, or the emergence of variants not fully covered by current vaccines. Similar patterns across West Africa, including Nigeria and Cameroon [95,96], highlight the need to optimize vaccination strategies to better meet regional- and community-specific needs. Position 426 differs among the three CPV strains, and has been suggested to contribute to antigenic drift, potentially affecting host antibody recognition [76]. The implications of these mutations for vaccine efficacy and immune protection will need to be investigated in future studies.

The detection of viruses in apparently healthy animals indicates the presence of subclinical infections and cryptic transmission cycles that may go undetected. Such circulation can still impact productivity, trade, and livelihoods, particularly in smallholder settings, and highlights persistent gaps in understanding host range, pathogenicity, and cross-species transmission. The presence of viruses across livestock, companion animals, and wildlife further emphasizes the permeability of ecological boundaries and the potential for spillover at human–animal interfaces. Addressing these gaps will require longitudinal, integrated surveillance combining genomic, ecological, and epidemiological data.

From a systems perspective, these findings support the integration of genomic surveillance into routine veterinary and One Health frameworks to move beyond pathogen detection toward a more complete understanding of viral ecology and emergence [97,98]. Such integration would improve early detection, enable more informed risk assessment, and guide targeted interventions, including vaccination strategies, biosecurity measures, and livestock import controls. Strengthening genomic capacity, in line with Ghana’s Joint External Evaluation recommendations, represents a practical pathway to more resilient, data-driven surveillance systems.

We were unable to revisit farms with positive viral detections for additional animal sampling or confirmatory testing due to logistical constraints, limited supplies, and labor limitations during the study period. Follow-up sampling of affected farms would help confirm infection status and provide a better understanding of viral prevalence, transmission dynamics, and associated disease outcomes. Metagenomic sequencing provides an invaluable, agnostic approach for capturing a broad spectrum of circulating pathogens; however, in samples with low viral titers, metagenomics often struggles to yield complete genomes, a challenge evidenced by the recovery of only segment 1 out of 11 segments for the *E. helvum* RVA strain in this study. Incomplete genome recovery restricts our ability to robustly assess complex evolutionary mechanisms, such as reassortment. Nevertheless, previous sequencing studies have demonstrated uneven recovery across RVA genome segments in NGS data, with segment 1 frequently exhibiting higher recovery rates, likely reflecting segment-specific amplification and sequencing biases [99]. Moving forward, complementing broad metagenomic screening with targeted genomic enrichment panels will be critical for improving the resolution of evolutionary tracking and better identifying strains with zoonotic potential. The cross-sectional design restricts inference on transmission dynamics, and the absence of linked human or serological data limits interpretation of zoonotic pathways. In addition, geographic and sampling constraints may not fully capture the diversity of circulating viruses. These limitations highlight the importance of longitudinal, multi-sectoral studies that integrate genomic, ecological, and epidemiological data across spatial and temporal scales.

The 2025 Joint External Evaluation of Ghana recognized the country’s proactive One Health approach and improved public health capacity since 2017, but noted limited capacity for zoonotic disease surveillance [11]. Within the context of Ghana’s Joint External Evaluation recommendations, this work provides a practical demonstration of how metagenomics-informed surveillance can strengthen national capacity for zoonotic disease preparedness and contribute to more resilient, data-driven health systems. Expanding such approaches across West Africa will be critical to improving early warning systems, guiding intervention strategies, and strengthening preparedness for future zoonotic threats.

## Figures and Tables

**Table 1:** Description of study animal samples. The number of animals sampled is given by region and by season.

	*B. taurus*	*C. familiaris*	*E. helvum*	*E. buettikoferi*	*E. franqueti*	*H. monstrosus*	*L. angolensis*	*N. veldkampii*	*S.s. domesticus*	*Total*
**Region**										
Ashanti	19	8	6	3	5		1	10	9	61
Bono East	5								6	11
Savanna		9								9
Volta									9	9
Western	19	25	2	19	2	1		1	7	76
*Total*	43	42	8	22	7	1	1	11	31	166
**Season**										
Dry	13	10							17	40
Rainy	30	32	8	22	7	1	1	11	14	126

**Table 2:** Description of viral sequences obtained in this study, including sampling and sequencing information.

Virus	Animal (ID)	Contig Name (# bp)	GenBank Accession	Sampling Region	BLAST Best Match (Accession)	Percent Identity (%)	E Value	Query Cover (%)	Average Coverage Depth	Genome Region
PPV3	Pig (P32)	k141_88414 (5208)	PX310535.1	Volta	MZ577031.1	99	~0	100	1352	Whole Genome
RVA	Bat (FK39)	k59_84 (3143)	PZ284986.1	Ashanti	KX268776.1	98	~0	100	30	Near Whole Segment 1
BovHepV	Cattle (Pool 6C)	k59_164 (8802)	PZ284983.1	Ashanti	NC_026797.1	91	~0	100	120	Whole Genome
CPV-2	Dog (Pool 1D)	k59_26528 (4953)	PZ284985.1	Savanna	PV784947.1	99	~0	100	607	Near Whole Genome
	Dog (Pool 3D)	k59_937 (5034)	PZ284984.1	Western	MF805796.1	99	~0	100	302	Whole Genome

## Data Availability

Raw sequencing data are submitted to SRA (SUB16128940), while viral genomes are deposited to GenBank under accession numbers: PX310535.1, PZ284983.1–PZ284986.1.

## Supplementary Materials

The following supporting information can be downloaded at: https://www.mdpi.com/article/10.3390/v18060644/s1, Table S1: Animal sample metadata; Table S2: GenBank accession numbers for reference datasets; Table S3: Search terms of NCBI search strategy; Table S4: Pairwise genetic distance analysis; Table S5: Estimated viral evolutionary rates; Figure S1: ML tree of PPV3; Figure S2: Capsid structure of PPV3; Figure S3: ML tree of CPV-2; Figure S4: Capsid structure of CPV-2; Figure S5: ML tree of BovHepV; Figure S6: Envelope structure of BovHepV; Figure S7: Expanded MCC tree of RVA segment 1 visualizing collapsed human sequences; Figure S8: ML tree of RVA segment 1; Figure S9: RdRp structure of RVA segment 1.

## Abbreviations

The following abbreviations are used in this manuscript:

BovHepV	Bovine hepacivirus
CPV-2	Canine parvovirus 2
HCV	Hepatitis C virus
HPDI	Highest posterior density interval
MCC	Maximum clade credibility
PPV3	Porcine parvovirus 3
RdRp	RNA-dependent RNA polymerase
RVA	Rotavirus A
VP1u	Viral protein 1 unique region
