# Supplementary material for: One Health Genomic Surveillance at Human–Animal Interfaces in Rural Ghana Reveals Underreported Viruses of Zoonotic and Economic Concern
Source: Viruses. 2026 Jun 3;18(6):644. doi: 10.3390/v18060644 (PMC13308164; doi:10.3390/v18060644)
Supplement: Supplementary file 1 [file viruses-18-00644-s001.zip › Supplemental Figures and Tables Legends.pdf]

**Supplemental Figures**

**Supplemental Figure 1.** Maximum likelihood tree of PPV3. Tips colored by country of origin. Black star indicates placement of study sequence PX310535.1 in tree. Diamonds at nodes indicate ultrafast bootstrap values above 90. Viruses labeled by GenBank accession number, location, and date. Scale bar in nucleotide substitutions per site. Outgroup rooted by Human Parvovirus 4 (KM390024.1), not shown for visualization purposes.

**Supplemental Figure 2.** The capsid proteins of PPV3. A) A schematic depiction of the overlapping VPs is shown with their size in amino acids (aa). Selected sequence motifs are indicated; RBD, Receptor binding domain; PLA2, Phospholipase A2; VP1u, VP1 unique. Below an AlphaFold3 model of the VP1u region is shown with the RBD, PLA2, N- and C-terminus labeled. The $\alpha$-helices are colored red. B) A radially colored surface representation of the PPV3 capsid is depicted generated from 60 icosahedrally arranged VP2 subunits. C) Maximum likelihood tree of the VP1 sequence for study sequence and the closely related Italian and Romanian isolates. Tips colored by country of origin. Animal symbols indicate origination from wild boar or domestic pig. D) An amino acid sequence comparison of the Ghanaian, Italian, and Romanian isolates is shown for the VP1u and VP2 region. Amino acid changes relative to the Ghanaian virus are highlighted in red and surface-exposed capsid residues are indicated with an asterisk.

**Supplemental Figure 3.** Maximum likelihood tree of CPV-2. Tips colored by continent of origin. Black star indicates placement of study sequences PZ284985.1 and PZ284984.1 in tree. The heatmap is colored by CPV-2 genotype. Diamonds at nodes indicate ultrafast bootstrap values above 90. Scale bar in nucleotide substitutions per site. Outgroup rooted by Feline panleukopenia virus (MZ712026.1), not shown for visualization purposes.

**Supplemental Figure 4.** The capsid proteins of CPV-2. A) Maximum likelihood tree of the VP1 sequence for study sequence and representative isolates of variants -2a and -2b. Tips colored by variant type. B) An amino acid sequence comparison of the Ghanaian, Hungarian, and Chinese isolates is shown for the VP1u and VP2 region. Amino acid changes relative to the Ghanaian virus are highlighted in red and surface-exposed capsid residues are indicated with an asterisk. VP2 numbering is utilized for residues within VP2.

**Supplemental Figure 5.** Maximum likelihood tree of BovHepV Genotype 1. Tips colored by country of origin. Black star indicates placement of study sequence PZ284983.1 in tree. The heatmap is colored by BovHepV subtype. Diamonds at nodes indicate ultrafast bootstrap values above 90. Scale bar in nucleotide substitutions per site. Outgroup rooted by Bat hepacivirus (NC_031947.1), not shown for visualization purposes.

**Supplemental Figure 6.** The envelope proteins, E1 and E2, of BovHepV. A) Maximum likelihood tree of the E1 and E2 sequences of BovHepV subtype B isolates sampled in Ghana, including study sequence PZ284983.1. B) AlphaFold predicted structure of the E1/E2 heterodimer for PZ284983.1. An amino acid sequence comparison of the Ghanaian subtype B isolates is shown for the E1 and E2 regions. Amino acid changes relative to PZ284983.1 are highlighted in red.

**Supplemental Figure 7.** Non-collapsed time-scaled maximum clade credibility (MCC) tree estimated from RVA segment 1 sequences. (A) MCC tree inferred using the uncorrelated relaxed clock and Hamiltonian Monte Carlo SkyGrid implemented in BEAST X. Tips are colored according to continent of origin. The heat maps are colored according to host and genotype. Black triangles at internal nodes indicate branches supported by posterior probability > 0.9. The genotype R16 subclade containing

sequence from Ghana generated in this study, PZ284986.1, is highlighted with a black star. (B) Zoomed view of the genotype R16 subclade shown in panel A. For selected nodes, *a* and *b*, the time to most recent common ancestor (TMRCA), and 95% highest posterior density (HPD) intervals are given. Node *a* represents the most recent common ancestor of our study sequences and *E. helvum* R16 sequences. Node *b* represents the common ancestor of study sequence PZ284986.1 and KX268776.1 sampled in Cameroon. Viruses are labelled by GenBank ID, host, and date of collection.

**Supplemental Figure 8.** Maximum likelihood tree of RVA segment 1. Tips colored by continent of origin. Black star indicates placement of study sequence PZ284986.1 in tree. The heatmap is colored by host on the left and genotype on the right. Diamonds at nodes indicate ultrafast bootstrap values above 90. Scale bar in nucleotide substitutions per site. Outgroup rooted by Rotavirus C segment 1 (NC_007547.1), not shown for visualization purposes.

**Supplemental Figure 9.** The RdRp protein of RVA. A) Maximum likelihood tree of the RdRp sequence of RVA genotype R16 isolates. Tips colored by country collection. B) AlphaFold predicted structure of RdRp for the study sequence, PZ284986.1. The regions of the RdRp are colored and labeled accordingly. An amino acid sequence comparison of the R16 isolates is shown, with substitutions in the N-terminal domain indicated in gray and those in the bracelet domain in orange. Amino acid changes relative to PZ284986.1are highlighted in red.

**Supplementary Tables**
**Supplementary Table 1.** Complete metadata of study samples.

**Supplementary Table 2.** List of accession numbers for virus datasets.

**Supplementary Table 3.** Search terms for generating virus datasets.

**Supplementary Table 4.** Pairwise genetic distance analysis for study viruses.

**Supplementary Table 5.** Estimated viral evolutionary rates.

**Supplemental Tables**
