# Supplementary figures and images for: One Health Genomic Surveillance at Human–Animal Interfaces in Rural Ghana Reveals Underreported Viruses of Zoonotic and Economic Concern
Source: Viruses. 2026 Jun 3;18(6):644. doi: 10.3390/v18060644 (PMC13308164; doi:10.3390/v18060644)

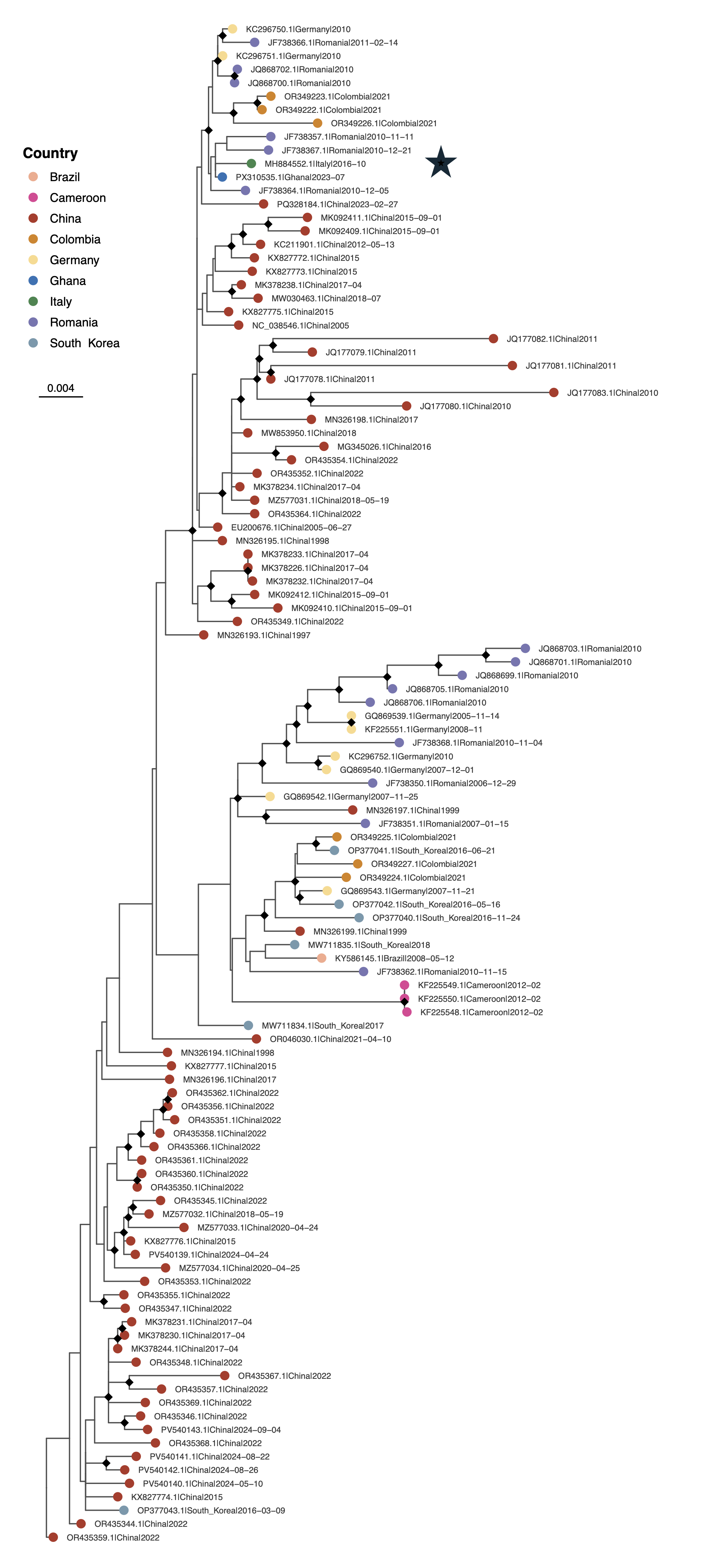

Supplement: Supplementary file 1 [file viruses-18-00644-s001.zip › Figure_S1_PPV3_ML.tiff]

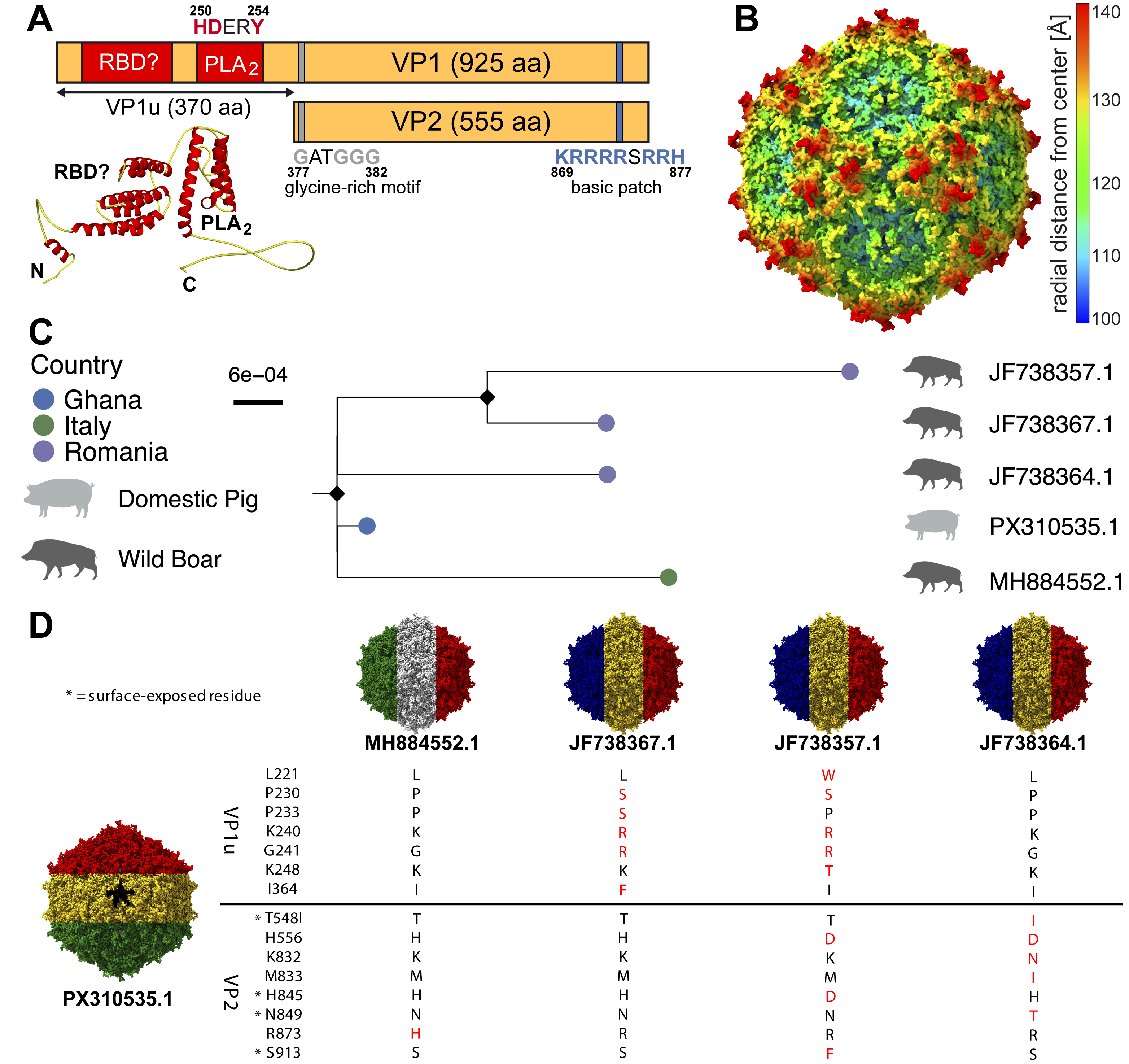

Supplement: Supplementary file 1 [file viruses-18-00644-s001.zip › Figure_S2_PPV3_capsid.tif]

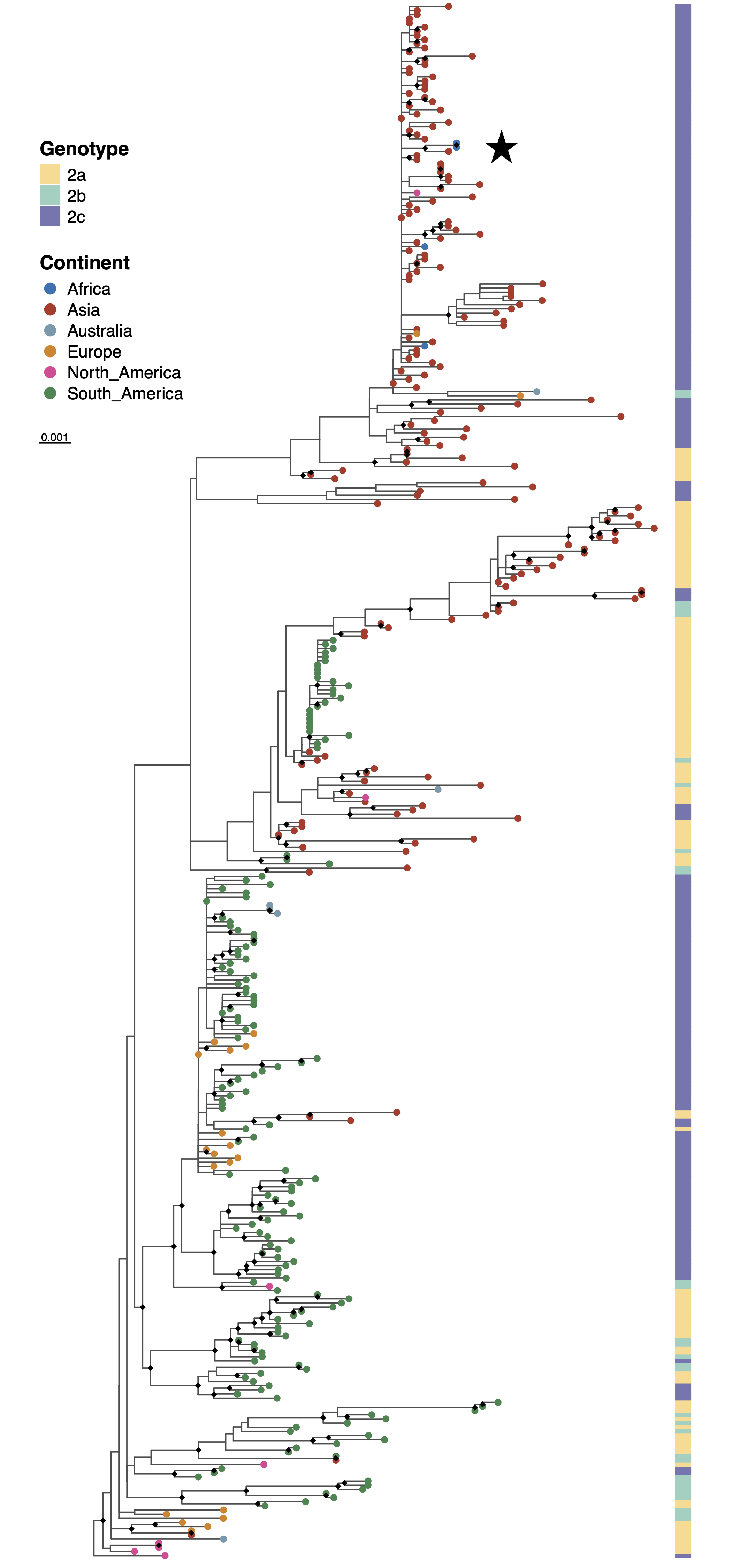

Supplement: Supplementary file 1 [file viruses-18-00644-s001.zip › Figure_S3_CPV_ML.tiff]

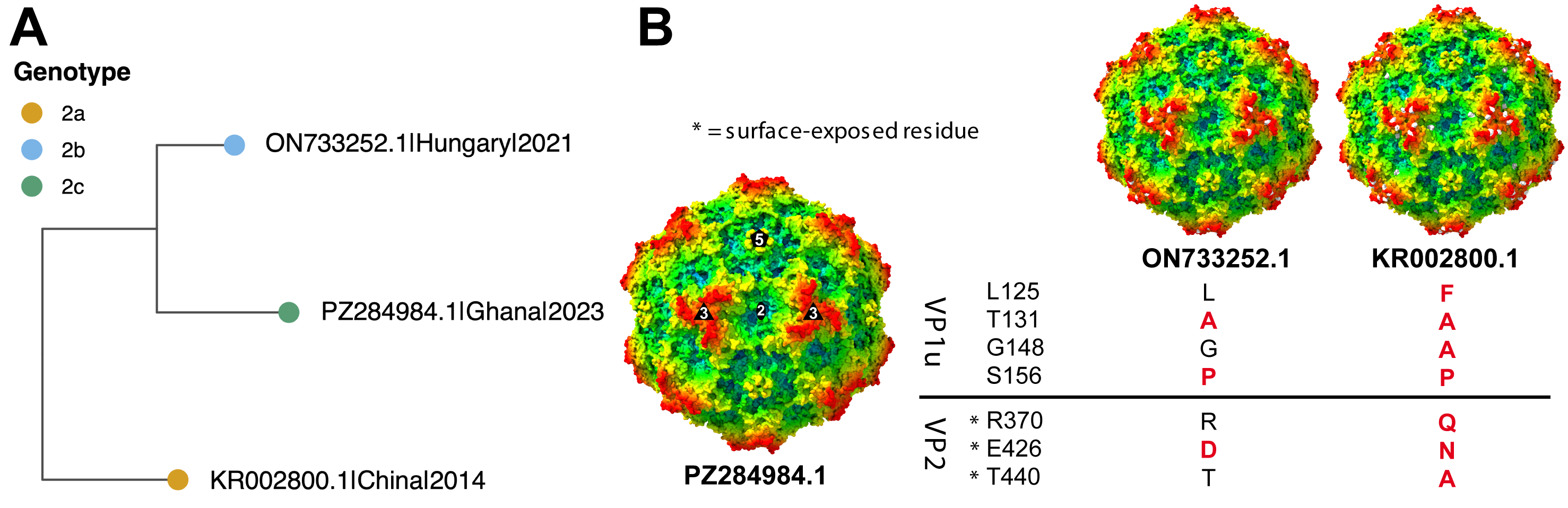

Supplement: Supplementary file 1 [file viruses-18-00644-s001.zip › Figure_S4_CPV_capsid.tif]

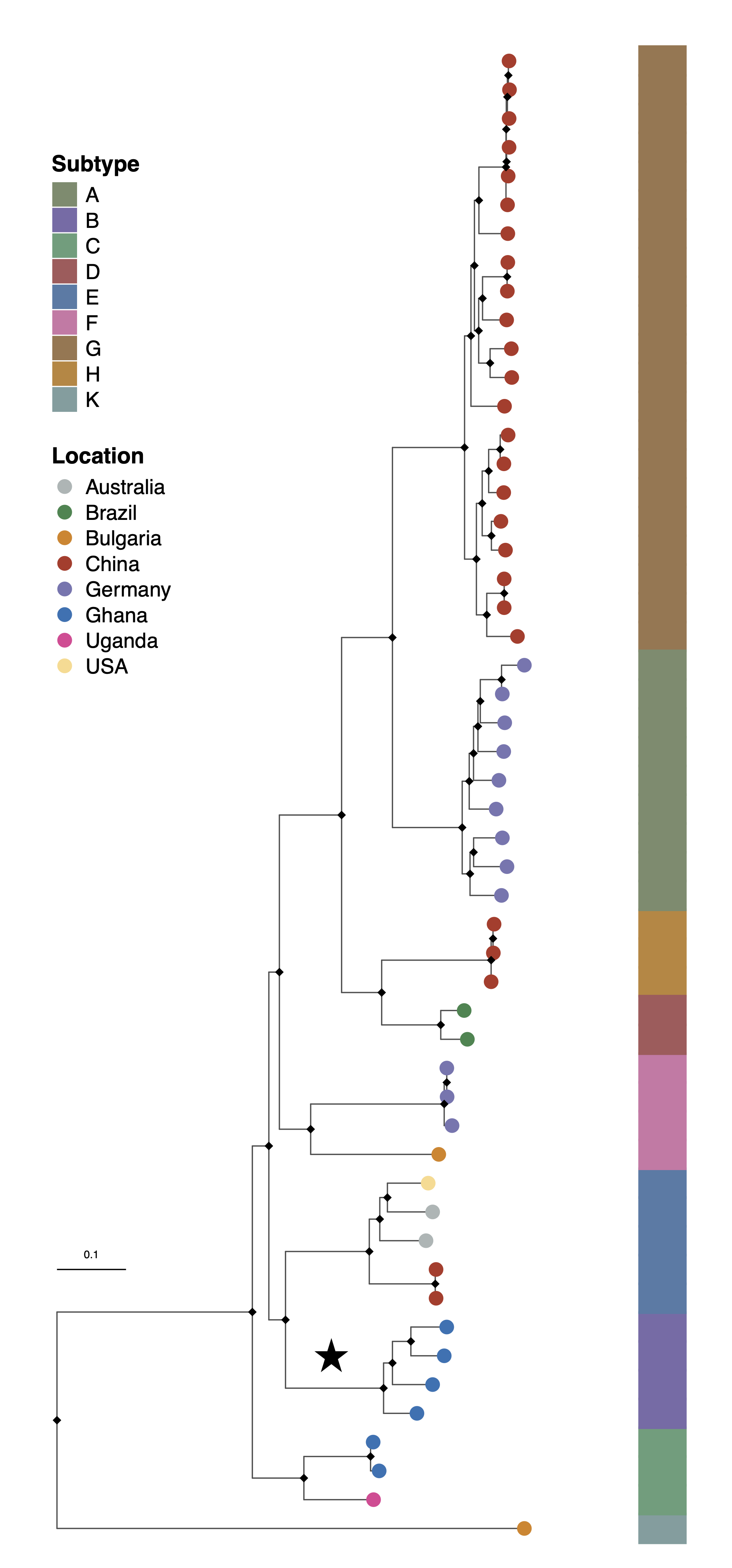

Supplement: Supplementary file 1 [file viruses-18-00644-s001.zip › Figure_S5_BovHepV_ML.tiff]

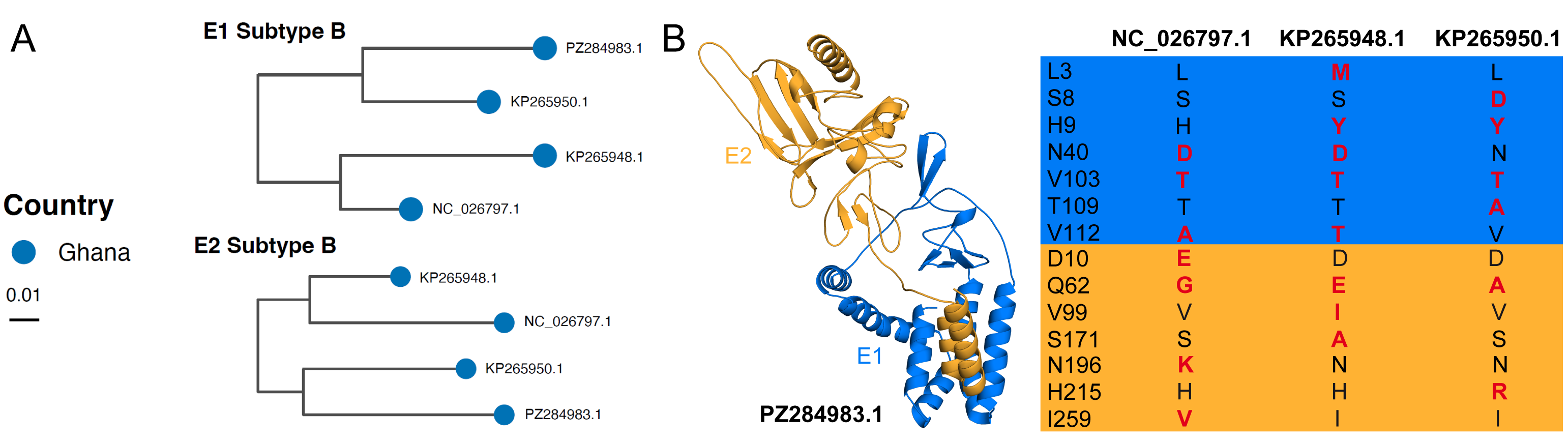

Supplement: Supplementary file 1 [file viruses-18-00644-s001.zip › Figure_S6_BovHepV_Envelope.tif]

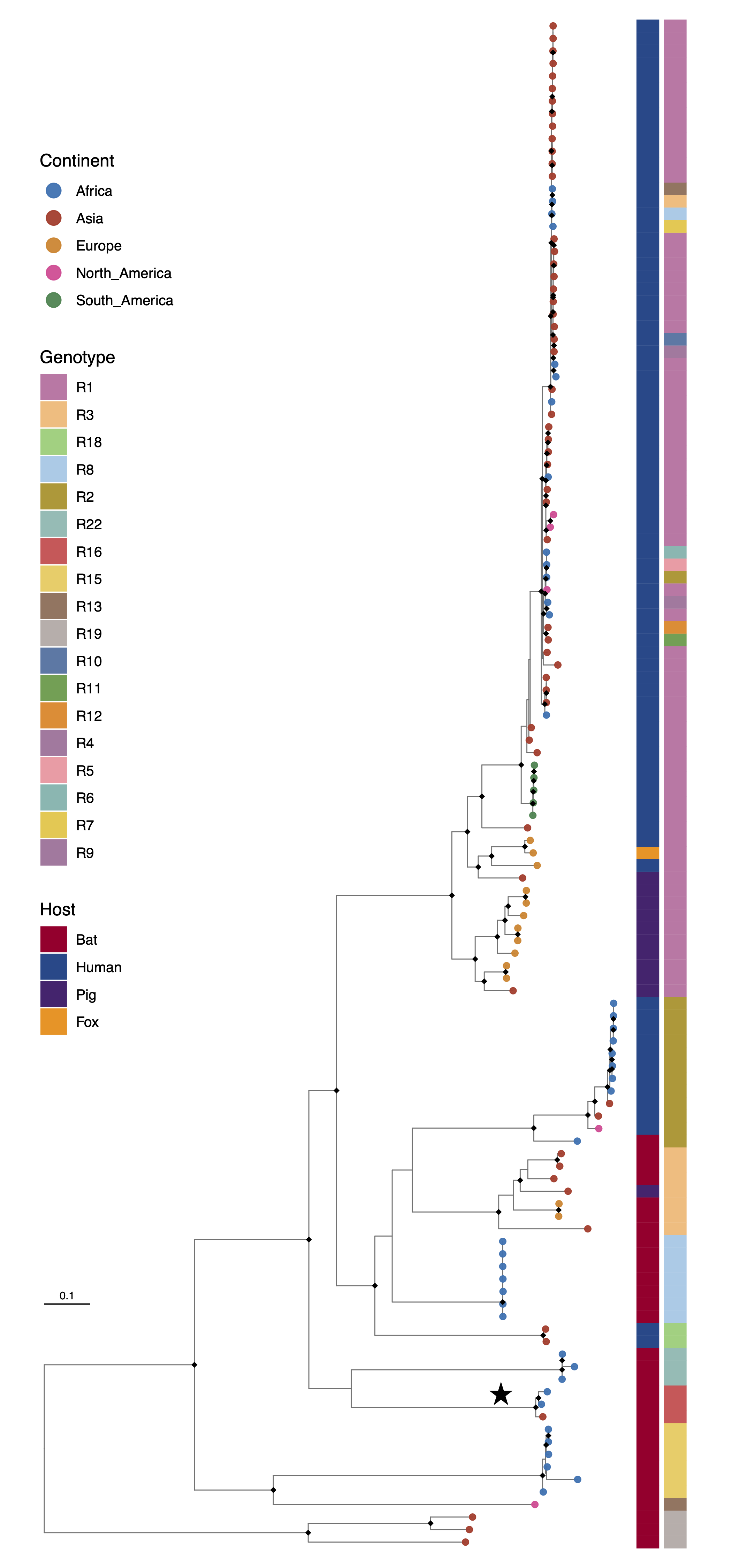

Supplement: Supplementary file 1 [file viruses-18-00644-s001.zip › Figure_S8_RVA_ML.tiff]

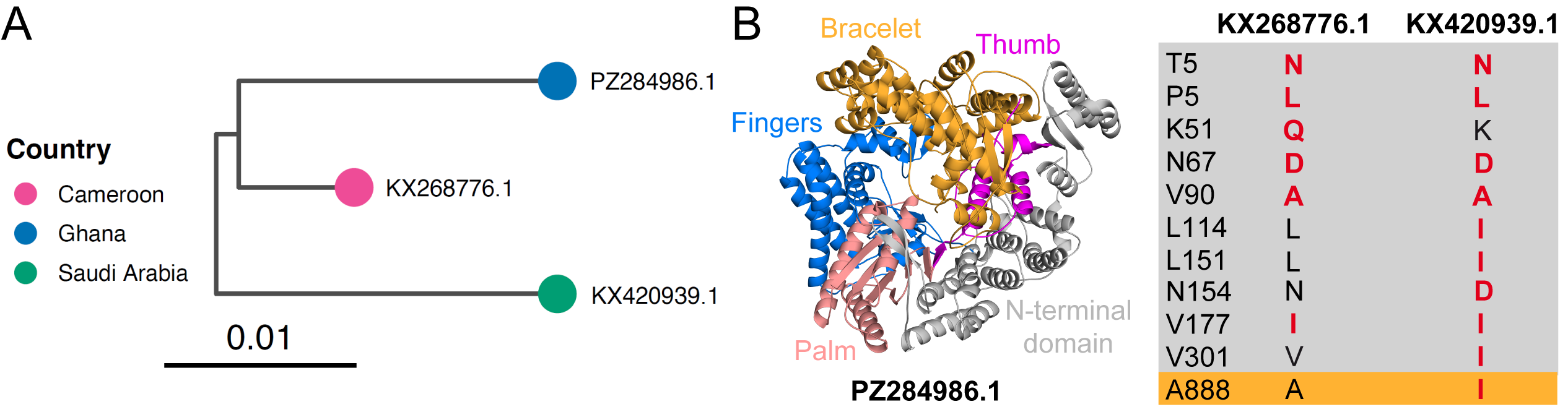

Supplement: Supplementary file 1 [file viruses-18-00644-s001.zip › Figure_S9_RVA_RdRp.tif]
